# Correction: Enhancing the multifunctional properties of polycaprolactone/chitosan films with zirconium dioxide nanoparticles for biomedical and flexible optoelectronic applications

**DOI:** 10.1039/d5ra90109j

**Published:** 2025-09-22

**Authors:** Qasim Shakir Kadhim, Zohra Benzarti, Mohanad H. Mousa, Maher Hassan Rasheed, Najmeddine Abdelmoula, Ali Khalfallah

**Affiliations:** a Department of Science, College of Basic Education, University of Babylon Babylon Iraq; b Laboratory of Multifunctional Materials and Applications (LaMMA), Faculty of Sciences of Sfax, University of Sfax BP 1171 3000 Sfax Tunisia zohra.benzarti@fss.usf.tn; c University of Coimbra, CEMMPRE, ARISE, Department of Mechanical Engineering Rua Luís Reis Santos Coimbra 3030-788 Portugal; d Shatrah Technical Institute, Southern Technology University (STU) Basra Iraq; e Department of Science, College of Basic Education, University of Babylon Babylon Iraq; f DGM, Institut Supérieur des Sciences Appliquées et de Technologie de Sousse, Université de Sousse Cité Ibn Khaldoun 4003 Sousse Tunisia

## Abstract

Correction for ‘Enhancing the multifunctional properties of polycaprolactone/chitosan films with zirconium dioxide nanoparticles for biomedical and flexible optoelectronic applications’ by Qasim Shakir Kadhim *et al.*, *RSC Adv.*, 2025, **15**, 31788–31805, https://doi.org/10.1039/D5RA05303J.

The authors regret that the name of one of the authors (Qasim Shakir Kadhim) was shown incorrectly in the original article. The corrected author list is as shown here.

The Royal Society of Chemistry apologises for these errors and any consequent inconvenience to authors and readers.

